# Defining minimal detectable difference in echocardiographic measures of right ventricular function in systemic sclerosis

**DOI:** 10.1186/s13075-022-02835-5

**Published:** 2022-06-18

**Authors:** Monica Mukherjee, Valentina Mercurio, Aparna Balasubramanian, Ami A. Shah, Steven Hsu, Catherine E. Simpson, Rachel Damico, Todd M. Kolb, Paul M. Hassoun, Stephen C. Mathai

**Affiliations:** 1 Divisions of Cardiology, Johns Hopkins University, 1830 E. Monument Street, Room 540, Baltimore, MD 21205 USA; 2 Division of Pulmonary and Critical Care Medicine, Johns Hopkins University, 1830 E. Monument Street, Room 540, Baltimore, MD 21205 USA; 3 Divison of Rheumatology, Johns Hopkins University, 1830 E. Monument Street, Room 540, Baltimore, MD 21205 USA

**Keywords:** Pulmonary hypertension, Systemic sclerosis, Echocardiography, Minimal detectable difference

## Abstract

**Background:**

Echocardiography (2DE) is integral for screening and longitudinal evaluation of pulmonary arterial hypertension (PAH) in systemic sclerosis (SSc). In the present study, we sought to establish the reliability, repeatability, and reproducibility of 2DE parameters in SSc patients with and without PAH and to define the minimal detectable difference (MDD), the smallest change detected beyond measurement error.

**Methods:**

SSc patients without known PAH and with invasively confirmed PAH on stable therapies underwent 2DE with strain at two time points. Analysis of variance (ANOVA) and coefficients of variation (CV) were calculated to assess for repeatability, reliability, and reproducibility. Intra- and inter-observer agreement were assessed using intraclass correlation. Bland-Altman analysis explored the level of agreement between evaluations. MDD was calculated using the standard error of measurement for each parameter by cohort.

**Results:**

ANOVA demonstrated few significant differences between evaluations across groups. Global right ventricular longitudinal systolic strain (GRVLSS, 9.7%) and fractional area change (FAC, 21.3%) had the largest CV, while tricuspid annular plane excursion (TAPSE), S’ wave, and right ventricular outflow track velocity time integral (RVOT VTI) were 0.87%, 3.2%, and 6.0%, respectively. Intra- and inter-observer agreement was excellent. MDD for TAPSE, FAC, S’ wave, RVOT VTI, GRVLSS, and RVSP were 0.11 cm, 0.03%, 1.27 cm/s, 0.81 cm, 1.14%, and 6.5 mmHg, respectively.

**Conclusions:**

We demonstrate minimal measurement error in clinically important 2DE-based measures in SSc patients with and without PAH. Defining the MDD in this population has important implications for PAH screening, assessment of therapeutic response, and sample size calculations for future clinical trials.

**Supplementary Information:**

The online version contains supplementary material available at 10.1186/s13075-022-02835-5.

## Background

Pulmonary arterial hypertension (PAH), a disease of the pulmonary vasculature that leads to right heart failure and death, commonly complicates systemic sclerosis (SSc) [1]. Given its high morbidity and mortality, current guidelines recommend screening for PAH in SSc patients [2]. The screening algorithms used for early detection of PAH in SSc rely upon two-dimensional echocardiography (2DE) which, despite well-described limitations, has high specificity and positive predictive value [2, 3]. In addition, 2DE is recommended as part of follow-up evaluation and for risk assessment [4]. Clinically relevant metrics that are frequently used for early detection, follow-up, and risk assessment include right ventricular systolic pressure (RVSP, an estimate of pulmonary artery systolic pressure (PASP) [5], tricuspid annular plane systolic excursion (TAPSE) [6, 7], tissue Doppler of the tricuspid annulus S’ velocity [8], and fractional area change (FAC) [9, 10]. In addition, measures of RV contractile function utilizing speckle-tracking echocardiography (STE) have demonstrated regional abnormalities in RV contractile function in SSc [11], as well as value in assessing response to therapy and predicting mortality [12, 13]. Despite compelling evidence supporting the clinical relevance of these echocardiographic measures, to our knowledge, no study has specifically defined the repeatability, reproducibility, reliability, or the minimal detectable difference (MDD), the smallest change in a measurement of interest that is greater than the within subject variability and measurement error, in SSc or other populations. Defining the MDD is vital to characterizing responsiveness of a measurement to an intervention and a critical knowledge gap in the assessment of outcome measures in SSc-PAH [14].

In the present study, we sought to assess the performance of echocardiographic measures of RV function to assess the repeatability, reproducibility, and reliability of echo-derived measures of RV function and define the MDD. We hypothesized that TAPSE would have the least measurement error based upon our prior experience in SSc populations [6]. This work was presented at the 2020 American Thoracic Society Scientific Sessions, Philadelphia, PA, in abstract form.

## Methods

### Patient population

Our study was approved by the Johns Hopkins Medicine Institutional Review Board. We prospectively enrolled prevalent SSc patients ≥18 years old from May 2017 to October 2018. All participants met the 1980 [15] and/or 2013 American College of Rheumatology classification criteria for SSc [16]. .The Johns Hopkins Scleroderma Center’s standard clinical practice is to perform annual pulmonary function testing (PFTs) and 2DE to screen for cardiopulmonary complications [17]. Patients with significant chronic obstructive or interstitial lung disease, portal hypertension, severe obstructive sleep apnea, left-sided heart failure, or chronic thromboembolic disease were excluded [18]. Ever-usage of medications such as disease-modifying drugs, calcium channel blockers, and PAH therapies (endothelin receptor antagonists, phosphodiesterase type 5 inhibitors, and prostacyclin analogs or receptor agonists) was recorded.

Patients without evidence of resting PH or RV dysfunction by 2DE defined as resting RVSP < 35 mmHg and TAPSE ≥ 1.6 cm, FAC ≥ 35%, and tissue Doppler S’ ≥ 9.5 cm/s were recruited as cohort 1. Consecutive SSc patients with right heart catheterization (RHC)-proven PAH who were clinically stable on PAH-directed therapies were recruited as cohort 2. PAH was defined by a mean pulmonary artery pressure (mPAP) > 20 mmHg and pulmonary vascular resistance (PVR) > 3 Wood units (WU) with pulmonary artery wedge pressure ≤ 15 mmHg based on 2018 revised 6th World Symposium on Pulmonary Hypertension definition [4].

### Echocardiographic acquisition and measurements

Echocardiograms were performed using Canon Artida Ultrasound Machine (Canon Healthcare, Testin, CA) with subjects in the left lateral decubitus position during image acquisition at 70–90 frames per second at end-expiration. 2DE-directed methods to obtain linear and volumetric measurements of the RV chamber in accordance with American Society of Echocardiography (ASE) guidelines [19]. Right atrial area (RAA) was estimated using volumetric area from the apical 4-chamber view. RV function was assessed using TAPSE, tissue Doppler S’ velocity of the lateral tricuspid annulus, and FAC [20]. Tricuspid regurgitant (TR) velocity was used to estimate RVSP and PASP using the modified Bernoulli equation and adding estimated RA pressure based on inferior vena cava dimension and collapsibility with sniff [21, 22]. Estimation of right ventricular-arterial coupling was assessed by the ratio of TAPSE:PASP [23].

2DE echocardiographic measures were obtained by a single certified cardiac sonographer at two time points, evaluation A and evaluation B, separated by 1 h in a semi-fasting state, to limit biologic variability. Two echocardiographers, blinded to patient information, timing (i.e., before/after one-hour fixed time interval), and clinical variables performed 2DE analysis using Synapse Cardiovascular Software (FUJIFILM Medical Systems, V4.0.8, USA).

STE-based longitudinal systolic strain analysis of the RV free wall was performed using commercially available strain software (Epsilon EchoInsight Version 3.1.0.3358, Milwaukee, WI). From the 4-chamber apical view, peak systolic longitudinal strain of the RV free wall was obtained by tracing endocardial borders in end-systolic still frames and manually adjusted to ensure adequate border delineation and segment thickness [24]. Peak longitudinal systolic strain was defined as the difference in shortening from the region of interest relative to original length, and expressed as a negative percentage. Global RVLSS was defined as the average of regional strain from the basal, midventricular, and apical RV free wall segments.

### Analytic approach

Categorical variables were expressed as absolute number and percentage. Continuous variables were expressed as mean ± standard deviation (SD) if normally distributed by the Shapiro-Wilk test and as median (interquartile range, IQR) if not normally distributed. To assess statistical differences in all echo measures between evaluation A and evaluation B, within disease group (cohort 1 and cohort 2), and overall, across both cohorts, ANOVA tests for normally distributed variables and Friedman tests for non-normally distributed variables were performed.

Comparisons between cohort 1 and cohort 2 were performed with independent sample *t*-tests or Mann-Whitney-Wilcoxon rank sum test as appropriate. Fisher exact test was used to analyze differences in categorical variables between cohorts. A *P*-value < 0.05 was considered as statistically significant.

All examinations were analyzed twice on two different days by the same physician (MM) blinded to cohort and timing of the echocardiogram to determine intraobserver agreement. Examinations were then analyzed by a second reader (VM), also blinded to cohort, timing, and previous interpretations to determine interobserver agreement.

Repeatability, defined as the assessment of repeated measures on the same patient by the same operator on the same device under ideal conditions, was determined by calculating the SD for each patient between measurement A and B with a coefficient of variation (CV) (defined as SD divided by mean value, expressed as a percentage) [25]. Bland-Altman analysis was performed to assess for the level of agreement in measures between evaluation A and evaluation B and exclude the presence of proportional bias between approaches [26]. Reproducibility, defined as variations in measurements made on a subject under changing conditions, was assessed by intraclass correlation coefficient (ICC) for interobserver reproducibility both within disease group (cohort 1 and cohort 2) and in the overall cohort [25]. Reliability, defined as the degree to which the variability of the measurement compares to the inherent or true variability between subjects, was assessed by ICC for intraobserver agreement both within disease group (cohort 1 and cohort 2) and in the overall cohort.

MDD, defined as the minimal change in a measurement that is greater than the within subject variability and measurement error, was calculated for individual subjects between the two different observations. MDD was calculated as standard error of the measurement (SEM) × 1.96 and is reported by cohort [27]. SEM was calculated as the SD of the differences between the two observations for all participants divided by the square root of the sample size. Pearson’s correlation coefficient was used to analyse the relationship between TAPSE and other 2DE-based measures of RV function after normality of data distribution was assessed. Statistical analysis was performed using the SPSS statistical package (SPSS Inc., Chicago, IL, USA, version 20).

## Results

### Patient population

A total of 20 patients were included (Table 1). Cohort 1 consisted of 10 SSc patients without PAH who were, on average, 60.9 ± 8.0 years of age, and mostly women (80%). Cohort 2 consisted of 10 SSc-PAH patients who were mostly women (80%) and on average 61.7 ± 8.9 years of age with WHO functional class 2 symptoms. Across groups, most SSc patients in our pooled cohort had the limited disease subtype. Further details or SSc-defining characteristics are shown in Table 1. Hemodynamics from the SSc with PAH group were consistent with PAH of moderate severity. Of note, several patients in the SSc without PAH cohort received PAH specific therapy for non-PAH indications: ERA and prostacyclin analogs for management of Raynaud’s phenomenon and digital ulcers; PDE5I for erectile dysfunction.

**Table 1:** Clinical and hemodynamic characteristics of the study population

Characteristic	Total SSc population *n* = 20	Cohort 1 SSc without PAH, *n* = 10	Cohort 2 SSc with PAH *n* = 10	*P* value
Age, years	61.3 ± 8.2	60.9 ± 8.0	61.7 ± 8.9	NS
Female sex, *n* (%)	16 (80%)	8 (80%)	8 (80%)	NS
Disease subtype				
Limited, *n* (%)	16 (80%)	8 (80%)	8 (80%)	NS
Diffuse, *n* (%)	4 (20%)	2 (20%)	2 (20%)	NS
Autoantibody subtype, *n* (%)				
Positive anti-Scl70	2 (10%)	0	2 (20%)	NS
Positive anti-centromere	5 (25%)	4 (40%)	1 (10%)	NS
Positive anti-RNA polymerase	0	0	0	–
Positive ANA, centromere pattern	8 (42.1%)	5 (50%)	3 (33%)	NS
Positive ANA, nucleolar pattern	10 (52.6%)	4 (40%)	6 (67%)	NS
Positive ANA, RNP	1 (5.3%)	1 (10%)	0	NS
Medications (ever-usage)				
Calcium channel blockade, *n* (%)	17 (85%)	8 (80%)	9 (90%)	NS
Azathioprine, *n* (%)	0	0	0	NS
Cychophosphamide, *n* (%)	0	0	0	−
D-Penicillamine, *n* (%)	0	0	0	−
Intravenous immunoglobulins, *n* (%)	0	0	0	−
Leflunomide, *n* (%)	0	0	0	−
Methotrexate, *n* (%)	0	0	0	−
Mycophenolate mofetil, *n* (%)	5 (25%)	1 (10%)	4 (40%)	**0.04**
Plaquenil, *n* (%)	6 (30%)	4 (40%)	2 (20%)	NS
Predisone, *n* (%)	6 (31.6%)	2 (22%)	4 (40%)	NS
Rituximab, *n* (%)	0	0	0	−
PAH Therapies (ever-usage)				
Endothelin receptor antagonist, *n* (%)	6 (31.6%)	1 (11.1%)	5 (50%)	NS
Phosphodiesterase type 5 inhibitors, *n* (%)	9 (47.4%)	1 (11.1%)	8 (80%)	**0.001**
Prostacyclin analogs/receptor agonists, *n* (%)	3 (15.8%)	1 (11.1%)	2 (20%)	NS
WHO FC I/II/III, *n*	11/6/3	9/1/0	2/5/3	**0.02**
FVC, %	86 ± 21	92 ± 14	79 ± 26	NS
6-min walking distance, m	NA	NA	397 ± 122	−
Right atrial pressure, mmHg	NA	NA	5 ± 4	−
Mean PAP, mmHg	NA	NA	36 ± 7	−
PCWP, mmHg	NA	NA	9 ± 4	−
Cardiac Index, l/min/m^2^	NA	NA	2.3 ± 0.7	−
PVR, WU	NA	NA	6.1 ± 2.7	−

Data are expressed as mean ± standard deviation for the continuous variables, and as absolute number and percentage for the discrete variables, as appropriate

*Abbreviations*: *SSc* systemic sclerosis, *PAH* pulmonary arterial hypertension, *ACR*American College of Rheumatology, *WHO FC* World Health Organization functional class, *FVC* forced vital capacity, *NA* not available or not applicable, *PAP* pulmonary arterial pressure, *PCWP* pulmonary capillary wedge pressure, *PVR* pulmonary vascular resistance, *WU* Wood unit

### Echocardiographic measures

All measures were obtained and available for interpretation and analysis, apart from one patient from cohort 2 with an inadequate TR Doppler signal. Severity of TR differed between cohorts, with 10% of cohort 1 with moderate or severe TR compared to 50% of cohort 2. Conventional 2DE and STE-derived data are described in Table 2. The ratio of TAPSE to PASP, a noninvasive marker of RV contractile response to load, is also reported [23].

**Table 2:** Conventional echocardiographic and speckle-tracking derived measures of the study population

Echocardiographic parameter	Total SSc population *n* = 20		Variance *P* value	Cohort 1 SSc without PAH, *n* = 10		Variance *P* value	Cohort 2 SSc with PAH *n* = 10		Variance *P* value
	A	B		A	B		A	B	
RA area, cm^2^	18.3 ± 4.7	18.4 ± 5.8	0.66	16.0 ± 2.3	15.5 ± 2.4	0.37	20.6 ± 5.5	21.3 ± 6.7	0.24
RV hypertrophy yes, *n* (%)	16 (80)	16 (80)	1	7 (70)	7 (70)	1	9 (90)	9 (90)	1
RVH, cm	0.7 ± 0.1	0.7 ± 0.1	0.79	0.6 ± 0.1	0.6 ± 0.1	0.85	0.8 ± 0.1	0.8 ± 0.1	0.73
RVIDD, cm	3.0 ± 0.5	3.0 ± 0.5	0.68	2.7 ± 0.4	2.7 ± 0.4	1	3.2 ± 0.4	3.3 ± 0.4	0.66
Mid RVOT, cm	3.0 ± 0.5	3.0 ± 0.5	0.46	2.7 ± 0.4	2.8 ± 0.4	**0.04**	3.2 ± 0.4	3.2 ± 0.6	0.71
Distal RVOT, cm	2.0 ± 0.3	2.0 ± 0.4	0.55	2.0 ± 0.2	2.0 ± 0.2	0.78	2.1 ± 0.4	2.1 ± 0.4	0.61
RV base, cm	4.1 ± 0.6	4.1 ± 0.7	0.62	3.7 ± 0.5	3.6 ± 0.5	0.43	4.4 ± 0.6	4.6 ± 0.6	0.12
RV mid, cm	2.7 ± 0.7	2.7 ± 0.7	0.58	2.3 ± 0.4	2.3 ± 0.5	0.78	3.1 ± 0.7	3.1 ± 0.7	0.56
RV length, cm	8.4 ± 1.1	8.6 ± 1.1	0.06	7.7 ± 0.6	8.0 ± 0.5	0.10	9.1 ± 1.1	9.3 ± 1.2	0.36
RVED area, cm2	21.6 ± 7.4	22.1 ± 7.9	0.37	17.2 ± 3.2	16.7 ± 2.8	0.61	26.0 ± 8.0	27.5 ± 7.7	0.25
RVES area, cm2	10.7 ± 5.3	11.5 ± 6.0	0.10	7.9 ± 2.6	8.1 ± 2.2	0.82	13.5 ± 5.9	14.8 ± 6.8	0.07
FAC, %	52.1 ± 11.0	50.4 ± 12.5	0.27	54.2 ± 9.7	52.3 ± 6.9	0.35	50.0 ± 12.3	48.5 ± 16.6	0.55
TAPSE, cm	2.0 ± 0.4	2.0 ± 0.5	0.61	2.0 ± 0.3	2.0 ± 0.3	0.26	2.0 ± 0.5	2.1 ± 0.6	0.13
S’ wave, cm/s	12.4 ± 3.0	12.6 ± 2.9]	0.54	14.0 ± 1.9	14.1 ± 1.6	0.63	10.7 ± 3.1	11.0 ± 3.2	0.72
RVOT VTI, cm	13.8 ± 3.5	13.6 ± 3.3	0.77	13.9 ± 2.7	14.8 ± 2.6	0.18	13.6 ± 4.3	12.4 ± 3.6	**0.02**
RAP, mmHg	5.5 ± 3.4	5.5 ± 3.4	1.00	4.0 ± 2.1	4.0 ± 2.1	1.00	3.6 ± 1.6	3.6 ± 1.6	1.00
TR V max, m/s	2.8 ± 0.8 (*n* = 19)	3.0 ± 0.9 (*n* = 19)	**0.03**	2.3 ± 0.4	2.4 ± 0.4	**0.03**	3.2 ± 0.7 (*n* = 9)	3.5 ± 0.8 (*n* = 9)	0.14
RVSP, mmHg	35.8 ± 18.4 (*n* = 19)	42.2 ± 20.2 (*n* = 19)	0.07	25.6 ± 9.3	28.7 ± 9.5	**0.02**	46.1 ± 19.2 (*n* = 9)	55.7 ± 19.2 (*n* = 9)	0.18
TAPSE:PASP, mm/mmHg	0.7 ± 0.4	0.6 ± 0.4	0.52	0.9 ± 0.4	0.8 ± 0.5	0.55	0.5 ± 0.2	0.8 ± 0.5	0.72
Basal RVLSS, %	− 26.1 ± 8.0	− 27.6 ± 9.0	0.20	− 28.0 ± 5.8	− 30.7 ± 7.2	0.11	− 24.1 ± 9.7	− 24.4 ± 9.8	0.86
Mid RVLSS, %	− 19.8 ± 5.9	− 21.0 ± 7.5	0.34	− 23.8 ± 3.1	− 26.2 ± 5.2	0.15	− 15.8 ± 5.3	− 15.7 ± 5.6	0.96
Apical RVLSS, %	− 11.6 ± 6.0	− 11.0 ± 5.4	0.64	− 13.6 ± 7.0	− 13.5 ± 6.6	0.95	− 9.0 ± 3.9	− 9.8 ± 4.6	0.46
Global RVLSS, %	− 19.0 ± 5.1	− 20.1 ± 5.7	0.13	− 21.7 ± 2.8	− 23.5 ± 3.8	0.11	− 16.3 ± 5.5	− 16.7 ± 5. 4	0. 64

Data are expressed as mean ± standard deviation for the continuous variables and as absolute number and percentage for the discrete variables, as appropriate

*Abbreviations*: *IVS* interventricular septum, *PW* posterior wall, *LVIDD* left ventricular internal dimension diastole, *LVIDS* left ventricular internal dimension systole, *LA* left atrial, *RA* right atrial, *RV* right ventricular, *RVH* RV hypertrophy, *RVIDD* right ventricular internal dimension diastole, *RVOT* right ventricular outflow tract, *VTI* velocity time integral, *RVED* right ventricular end-diastolic, *RVES* right ventricular end-systolic, *FAC* fractional area change, *TAPSE* tricuspid annular plane systolic excursion, *S’* Tissue Doppler-derived right ventricular systolic excursion velocity, *PAEDV* pulmonary artery end-diastolic pressure, *PAEDP* pulmonary artery end-diastolic pressure, *RAP* right atrial pressure, *TR* tricuspid regurgitation, *RVSP* right ventricular systolic pressure, *PVR* pulmonary vascular resistance, *WU* Woods Unit, *RVLSS* right ventricular longitudinal systolic strain. RVSP and PASP are defined by the modified Bernoulli equation (4*(peak TR velocity)^2^ + RAP)

### Repeatability and agreement

After determining normal distribution for both cohort 1 (SSc without known PAH) and cohort 2 (SSc-PAH), ANOVA was performed to compare measures between evaluation A and evaluation B for each subject within each cohort and demonstrated no significant differences in echocardiographic parameters of RV morphology and function between evaluations in both groups, with the exception of midventricular RVOT diameter, RVOT VTI, TR peak velocity, and RVSP (*P* < 0.05) as shown in Table 2.

Table 3 details the SD and CV of repeated measures between evaluations within the same subject. SD of repeated measures were the lowest for TAPSE, FAC, and tissue Doppler S’, especially for cohort 1. SD of repeated measures were also low for RVOT VTI and global RVLSS but were lower for subjects in cohort 2 compared to cohort 1, suggesting less variability of these measures in the PAH cohort. SD of repeated measures between evaluations were the highest for RVSP regardless of cohort (14.3; 5.3; 24.2 for total SSc population, cohort 1, and cohort 2, respectively). As also shown in Table 3, CV was the lowest for TAPSE (0.9%; 0.6%; 1.1% for total SSc population, cohort 1, and cohort 2, respectively), TAPSE:PASP (1.7%; 2.2%; 0.6%), S’ wave (3.2%; 2.5%; 4.2%), RVOT VTI (6.0%; 6.9%; 5.0%), and global RVLSS (9.7%; 11.7%; 6.6%), while FAC (21.3%; 17.0%; 27.1%) and RVSP (38.0%; 19.6%; 48.6%) showed the highest values.

**Table 3:** Standard deviation and coefficient of variation of repeated echocardiographic measures by PAH status

Parameter	Standard deviation of repeated measures			Coefficient of variation (%)		
	Total SSc population	Cohort 1 (SSc without PAH)	Cohort 2 (SSc with PAH)	Total SSc population	Cohort 1 (SSc without PAH)	Cohort 2 (SSc with PAH)
**TAPSE, cm**	0.02	0.01	0.02	0.9	0.6	1.1
**FAC, %**	11.2	9.1	13.4	21.3	17.0	27.1
**Tissue Doppler S’**	0.4	0.3	0.5	3.2	2.5	4.2
**RVOT VTI, cm**	0.8	1.0	0.7	6.0	7.0	5.0
**Global RVLSS, %**	1.9	2.6	1.1	9.7	11.7	6.6
**RVSP, mmHg**	14.3	5.3	24.2	38.0	19.6	48.6
**TAPSE:PASP, mm/mmHg**	0.01	0.02	0.003	1.7	2.2	0.6

*Abbreviations*: *SSc* systemic sclerosis, *PAH* pulmonary arterial hypertension, *TAPSE* tricuspid annular plane systolic excursion, *FAC* fractional area change, *S’* Tissue Doppler-derived right ventricular systolic excursion velocity, *RVOT* right ventricular outflow tract, *VTI* velocity time integral, *RVLSS* right ventricular longitudinal systolic strain, *RVSP* right ventricular systolic pressure, *PASP* pulmonary artery systolic pressure

**Fig. 1:**
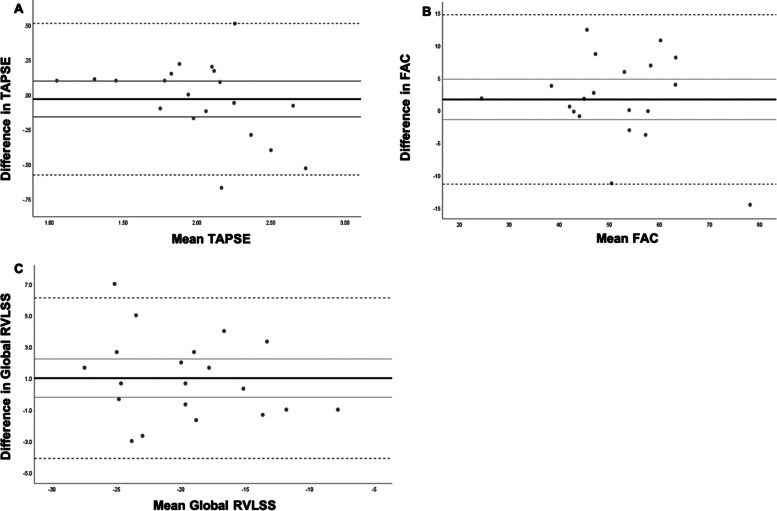
Bland-Altman analysis of agreement between Evaluation A and Evaluation B for tricuspid annular plane systolic excursion (TAPSE), fractional area change (FAC, %) and global right ventricular longitudinal strain (RVLSS). The black line represents the mean of the differences between Evaluation A and Evaluation B. The grey dashed lines represent the 95% confidence interval (CI). **A**: The black line represents the mean of the differences between TAPSE at Evaluation A and Evaluation B. The grey dashed lines represent the 95% CI (0.51071 and -0.57771 respectively). Unstandardized beta coefficient -0.282, *P*=0.06 (no proportional bias). **B**: The black line represents the mean of the differences between FAC at Evaluation A and Evaluation B. The grey dashed lines represent the 95% CI (14.7392 and -11.3522 respectively). Unstandardized beta coefficient -0.139, *P*=0.316 (no proportional bias); **C**: The black line represents the mean of the differences between global RVLSS at Evaluation A and Evaluation B. The grey dashed lines represent the 95% CI (6.10929 and -4.10929 respectively). Unstandardized beta coefficient -0.110, *P*=0.348 (no proportional bias)

Bland-Altman analysis for agreement by reader revealed no significant proportional bias for TAPSE, FAC, and global RVLSS (Fig. 1).

### Reliability

Reliability as assessed by intra-observer variability was excellent across the total population (Table 4, panel A). In cohort 1, ICC was excellent, defined as by ICC > 0.9, for FAC (0.930; 95% CI 0.816–0.973), tissue Doppler S’ velocities (0.975; 95% CI 0.937–0.990), global RVLSS (0.967; 95% CI 0.917–0.987), and RVSP (0.950; 95% CI 0.950–0.992) [28]. ICC was good for TAPSE and RVOT VTI at 0.75 and 0.9 [28]. In cohort 2, ICC was excellent for TAPSE (0.970; 95% CI 0.926–0.988), FAC (0.952; 95% CI 0.880–0.981), RVOT VTI (0.963; 95% CI 0.900–0.986), global RVLSS (0.950; 95% CI 0.775–0.990), and RVSP (0.993; 95% CI 0.983–0.997) and good for tissue Doppler S’ velocity. ICC for TAPSE to PASP was not determined since it is a derived measure.

**Table 4:** Intra- and inter-observer variability

Parameter	Panel A: Intra-observer variability			Panel B: Inter-observer variability		
	Total SSc population	Cohort 1 (SSc without PAH)	Cohort 2 (SSc with PAH)	Total SSc population	Cohort 1 (SSc without PAH)	Cohort 2 (SSc with PAH)
	ICC (95% CI)	ICC (95% CI)	ICC (95% CI)	ICC (95% CI)	ICC (95% CI)	ICC (95% CI)
TAPSE, cm	0.949 (0.903–0.973)**	0.891 (0.730–0.956)	0.970 (0.926–0.988)**	0.904 (0.871–0.949)**	0.767 (0.409–0.908)**	0.940 (0.848–0.976) **
FAC, %	0.947 (0.900–0.972)**	0.930 (0.816–0.973)	0.952 (0.880–0.981)**	0.854 (0.563–0.938)**	0.719 (0.121–0.899)**	0.891 (0.660–0.960) **
S’ wave, cm/s	0.903 (0.816–0.948)**	0.975 (0.937–0.990)	0.823 (0.552–0.930)**	0.975 (0.954–0.987)**	0.912 (0.777–0.965)**	0.980 (0.950–0.992) **
RVOT VTI, cm	0.941 (0.889–0.969)**	0.891 (0.722–0.957)	0.963 (0.900–0.986)**	0.873 (0.760–0.933)**	0.655 (0.143–0.863)*	0.940 (0.850–0.976) **
Global RVLSS, %	0.925 (0.692–0.982)**	0.967 (0.917–0.987)	0.950 (0.775–0.990)**	0.978 (0.959–0.989)**	0.938 (0.843–0.976)**	0.980 (0.949–0.992) **
RVSP, mmHg	0.994 (0.989–0.997)**	0.950 (0.950–0.992)	0.993 (0.983–0.997)**	0.990 (0.981–0.995)**	0.965 (0.912–0.986)**	0.986 (0.966–0.995) **

*Abbreviations*: *ICC* intraclass coefficient correlation, *CI* confidence interval, *TAPSE* tricuspid annular plane systolic excursion, *FAC* fractional area change, *S’* tissue Doppler-derived right ventricular systolic excursion velocity, *RVOT* right ventricular outflow tract, *VTI* velocity time integral, *RVLSS* right ventricular longitudinal systolic strain, *RVSP* right ventricular systolic pressure

^*^P-value< 0.01

^**^P-value< 0.001

### Reproducibility

Reproducibility as assessed by inter-observer variability was excellent across the pooled cohort for TAPSE, tissue Doppler S’ velocity, global RVLSS, and RVSP and good for both FAC and RVOT VTI (Table 4, panel B). By group, ICC was excellent for RVSP across cohort 1 (0.965; 95% CI 0.912–0.986) and cohort 2 (0.986; 95% CI 0.966–0.995) and for TAPSE (0.940; 95% CI 0.848–0.976), tissue Doppler S’ (0.980; 95% CI 0.950–0.992), and global RVLSS (0.980; 95% CI 0.949–0.992) in cohort 2. The lowest interobserver ICC agreements were observed for TAPSE, FAC, and RVOT VTI in the SSc patients without PAH. ICC for TAPSE to PASP was not determined since it is a derived measure.

### Minimal detectable difference

The MDD in the overall population for TAPSE was 0.11 cm, FAC 2.9%, tissue Doppler S’ velocity 1.3 cm/s, and global free wall RVLSS 1.1%, Table 5. Notably, the absolute MDD values were higher for TAPSE, FAC, and RVSP in cohort 2 (SSc-PAH) compared to cohort 1 (SSc without PAH). MDD for the TAPSE:PASP ratio, an echo-derived estimation of ventriculo-arterial coupling, was 0.1 mm/mmHg across the pooled cohort and higher at 0.16 mm/mmHg in the SSc without PAH group compared to 0.11 mm/mmHg in the SSc with PAH group.

**Table 5:** Minimum detectable difference for each echocardiographic measure of right ventricular function

Parameter	Minimal detectable difference		
	Total SSc population	Cohort 1 (SSc without PAH)	Cohort 2 (SSc with PAH)
**TAPSE, cm**	0.1	0.1	0.2
**FAC, %**	2.9	4.0	5.0
**S’ wave, cm/s**	1.3	0.8	1.0
**RVOT VTI, cm**	0.8	1.2	0.8
**Global RVLSS, %**	1.1	1.8	1.3
**RVSP, mmHg**	6.5	2.2	6.3
**TAPSE to PASP, mm/mmHg**	0.1	0.16	0.11

*Abbreviations*: *TAPSE* tricuspid annular plane systolic excursion, *FAC* fractional area change, *S’* tissue Doppler-derived right ventricular systolic excursion velocity, *RVOT* right ventricular outflow tract, *VTI* velocity time integral, *RVLSS* right ventricular longitudinal systolic strain, *RVSP* right ventricular systolic pressure, *PASP* pulmonary artery systolic pressure

## Discussion

In the present study, we sought to define the performance of echocardiographic measures of RV function in SSc patients with and without PAH. Under rigorous study conditions, we demonstrate high degrees of reproducibility, reliability, and repeatability for most measures. Bland-Altman analysis for agreement by reader revealed no significant proportional bias for TAPSE, FAC, and global RVLSS. Importantly, we define the MDD of RV functional measures for SSc patients with and without PAH. To our knowledge, this is the first study to establish key characteristics of echocardiographic measures of RV function. Furthermore, although our study included only SSc patients, our findings are likely applicable to other forms of PAH and thus have important implications for evaluation and management of this disease.

Echocardiography is integral in the screening for cardiopulmonary complications in SSc as well as serial monitoring of disease progression and treatment response in SSc-PAH [5]. However, despite its widespread use, no echocardiographic measures of RV function have been fully validated, as noted by the Expert Panel on Outcome Measures in PAH-SSc [14]. This lack of validation represents a major knowledge gap in the non-invasive assessment of RV function [29]. In the current study, we define the reproducibility, reliability, and repeatability of several echocardiographic measures of RV function to address this gap and to fulfil imaging standards defined as essential components for quality assurance and appropriate integration into study design and clinical trial analysis [30, 31]. We employed techniques to limit variability across various study aspects including technical components, uniformity of ultrasound equipment and analytical software, utilization of a single sonographer trained in acquisition of echocardiographic data for clinical trials, oversight of image acquisition and quality assurance, and analysis by two expert echocardiographers blinded to clinical variables and timing of 2DE examination to limit inter- and intra-observer variability [30]. In addition, echocardiograms were performed in a semi-fasting state within one hour to further limit biological variability. Thus, our study meets the imaging standards necessary to define measurement characteristics of RV function in echocardiography.

Our study demonstrates excellent repeatability as assessed by CV for most echocardiographic measures in both the SSc without and SSc with PAH groups. However, in the SSc-PAH group (cohort 2), both FAC (CV 27.08%) and RVSP (48.61%) had CVs that exceed commonly used thresholds for acceptable variability of 15–20%, suggesting poor repeatability of these measures [32]. Our data shows good-to-excellent levels of reliability and reproducibility for all parameters, based on ICC values for inter- and intra-observer agreement, though greater variability in most measures were noted in SSc-PAH patients compared to SSc patients without PAH. Differences in the variability of non-invasive measures of RV function between healthy controls and PAH patients have previously been demonstrated in a study of cardiac magnetic resonance imaging [33] and may be explained by physiologic adaptations of increasing RV end-systolic and end-diastolic volumes to maintain stroke volume in response to increased RV afterload. This leads to increases in RV dilatation which would necessarily cause increased variability in echo-based measures of RV function in PAH patients in whom RV afterload is elevated [34]. Interestingly, the lowest interobserver ICC agreements were observed for TAPSE, FAC, and RVOT VTI in the SSc patients without PAH and may suggest decreased sensitivity of these non-invasive parameters at lower afterloads.

Prior studies in PAH populations (not solely comprised of SSc-PAH) have not routinely reported ICC as a measure of reproducibility or reliability; thus, direct comparison to these studies is challenging. Hinderliter et al. reported the reproducibility of select measures in a PAH population from a randomized controlled trial of epoprostenol by comparing the repeated interpretations of a selection of 17 baseline echocardiograms [35]. The difference (mean ± SE) between the two interpretations for echo-based measurements obtained in that study was: 1.4 ± 0.2 cm^2^/m for indexed RV end-diastolic area; 4.7 ± 0.1% for FAC; and 0.08 ± 0.01 m/s for peak TR velocity [31]. Similarly, Nath and colleagues reported the reproducibility of RV size and RV function by comparing interpretations of 10 subjects’ echocardiograms who were randomly selected from cohort study of PAH patients and found the interobserver agreement for RV size was 80% and RV function was 70% [36]. Unfortunately, neither details regarding the metrics used to define RV size and RV function nor the method by which reproducibility was calculated were presented. Furthermore, as echocardiographic parameters that integrate the RV contractile response to pulmonary vascular load such as TAPSE to PASP have emerged as important predictors of PAH in SSc [23, 37, 38], it is increasingly important to define repeatability, reproducibility, and reliability. To our knowledge, no other study has rigorously evaluated other aspects of test characteristics for echocardiographic measures of RV function.

A key and novel component of the present study is the identification of the MDD for echocardiographic RV functional measures. Although not equivalent to the minimum clinically important difference, an MDD represents a key reference point upon which the lower bound of clinically relevant changes can be estimated [39, 40]. The MDD provides a framework for understanding if an observed change in a measure is related to inherent variability of the measure or if it represents real change from baseline. To put our MDD estimates in a clinical context, we reviewed several clinical studies that focused on the role of echocardiography in screening for PAH in SSc and assessing response to pulmonary vasodilator therapy in SSc-PAH patients. In a single observational study of 277 SSc patients unselected for PAH in whom changes in echo-based RV measures were assessed over a median follow-up of 3.3 years, the investigators found that an average decline in TAPSE of 0.14 cm, decrease in FAC of 1%, and increase in RVSP of 6 mmHg was associated with increased mortality [5]. The changes in TAPSE and RVSP associated with clinical outcomes in this study exceed the MDD for these measures as estimated by our current study; however, the change in FAC is significantly lower than the estimated MDD found in the current study, suggesting that changes in this range (1%) are within the range of measurement error and do not represent true change. In studies of various populations of PAH patients examining improvements in RV functional measures with pulmonary vasodilator therapy, changes in TAPSE of 0.2–0.56 cm and changes in FAC of approximately 3% were observed [41–43]. In an open-label clinical trial of combination oral therapy for treatment-naïve SSc-PAH patients, we have previously showed improvement in TAPSE by 0.55 cm, FAC by 11.8%, global RVLSS by 4.8%, and TAPSE:PASP 0.36 ± 0.24 over 36 weeks [13, 44]. The magnitude of the observed changes in each of these studies greatly exceed the MDDs reported in the current study, thereby confirming these changes as potentially clinically relevant.

There are several limitations to the present study. First, the relatively small sample size may influence the robustness of the reported measurement characteristics and thus require confirmation in larger cohorts. However, we did perform bootstrap analyses of the MDD calculations to estimate a population MDD with confidence intervals. These analyses show that the bootstrap-estimated population MDD and our study sample MDD are similar and, importantly, that the confidence intervals are narrow and thus consistent with the presented estimates of MDD (Supplement Table 1). This suggests that a larger sample size would be unlikely to yield difference results. Second, cohorts were frequency matched by age and gender; however, the predominance of women may impact the generalizability of our findings. Third, while we attempted to control for biovariability by conducting the study in a semi-fasting state at a fixed 1-h time interval, there may have been unanticipated biological factors that affected our findings. We also did not control for scleroderma disease duration and medications in our cross-sectional study design. Lastly, some measures of RV function, such as eccentricity index, were not included as part of our study protocol.

## Conclusions

In conclusion, these data on the variability of echo-based measures of RV function in SSc patients are highly relevant to the use and interpretation of these measures. The MDD for these measures offer an essential framework upon which estimation of clinically relevant changes can based to inform clinical decision-making, such as referral for RHC and escalation of therapies, not only in SSc but also in other forms of PAH. Further prospective studies are needed to establish the role of these echocardiographic measures in the management of SSc patients at-risk for and with known PAH and for patients with other forms of PAH.

## Supplementary Information

**Additional file 1: Supplemental Table 1.** Bootstrap estimated population minimal detectable differences for echocardiographic measurements.

## Data Availability

The datasets used and/or analyzed during the current study are available from the corresponding author on reasonable request.

## Abbreviations

2DE: 2D Echocardiography/echocardiogram
PAH: Pulmonary arterial hypertension
SSc: Systemic sclerosis
MDD: Minimal detectable difference
ANOVA: Analysis of variance
CV: Coefficients of variation
GRVLSS: Global right ventricular longitudinal systolic strain
TAPSE: Tricuspid annular plane systolic excursion
RVOT: Right ventricular outflow track
VTI: Velocity time integral
RV: Right ventricle
RVSP: Right ventricular systolic pressure
PAP: Pulmonary arterial pressure
PASP: Pulmonary arterial systolic pressure
FAC: Fractional area change
STE: Speckle-tracking echocardiography
RHC: Right heart catheterization
PFT: Pulmonary function testing
mPAP: Mean pulmonary arterial pressure
PVR: Pulmonary vascular resistance
WU: Wood units
ASE: American Society of Echocardiography
RAA: Right atrial area
TR: Tricuspid regurgitation
IQR: Interquartile range
SD: Standard deviation
ICC: Intraclass correlation coefficient
SE: Standard error

## References

[1] Steen VD, Medsger TA (2007). Changes in causes of death in systemic sclerosis, 1972-2002. Ann Rheum Dis.

[2] Coghlan JG, Denton CP, Grunig E, Bonderman D, Distler O, Khanna D (2014). Evidence-based detection of pulmonary arterial hypertension in systemic sclerosis: the DETECT study. Ann Rheum Dis.

[3] Galie N, Humbert M, Vachiery JL, Gibbs S, Lang I, Torbicki A (2015). 2015 ESC/ERS Guidelines for the diagnosis and treatment of pulmonary hypertension: The Joint Task Force for the Diagnosis and Treatment of Pulmonary Hypertension of the European Society of Cardiology (ESC) and the European Respiratory Society (ERS): Endorsed by: Association for European Paediatric and Congenital Cardiology (AEPC), International Society for Heart and Lung Transplantation (ISHLT). Eur Respir J.

[4] Simonneau G, Montani D, Celermajer DS, Denton CP, Gatzoulis MA, Krowka M, et al. Haemodynamic definitions and updated clinical classification of pulmonary hypertension. Eur Respir J. 2019;53:1801913. 10.1183/13993003.01913-2018 PMC6351336 30545968

[5] Shah AA, Chung SE, Wigley FM, Wise RA, Hummers LK (2013). Changes in estimated right ventricular systolic pressure predict mortality and pulmonary hypertension in a cohort of scleroderma patients. Ann Rheum Dis.

[6] Mathai SC, Sibley CT, Forfia PR, Mudd JO, Fisher MR, Tedford RJ (2011). Tricuspid annular plane systolic excursion is a robust outcome measure in systemic sclerosis-associated pulmonary arterial hypertension. J Rheumatol.

[7] Forfia PR, Fisher MR, Mathai SC, Housten-Harris T, Hemnes AR, Borlaug BA (2006). Tricuspid annular displacement predicts survival in pulmonary hypertension. Am J Respir Crit Care Med.

[8] Haeck ML, Scherptong RW, Marsan NA, Holman ER, Schalij MJ, Bax JJ (2012). Prognostic value of right ventricular longitudinal peak systolic strain in patients with pulmonary hypertension. Circ Cardiovasc Imaging.

[9] Raymond RJ, Hinderliter AL, Willis PW, Ralph D, Caldwell EJ, Williams W (2002). Echocardiographic predictors of adverse outcomes in primary pulmonary hypertension. J Am Coll Cardiol.

[10] Brierre G, Blot-Souletie N, Degano B, Tetu L, Bongard V, Carrie D (2010). New echocardiographic prognostic factors for mortality in pulmonary arterial hypertension. Eur J Echocardiogr.

[11] Mukherjee M, Chung SE, Ton VK, Tedford RJ, Hummers LK, Wigley FM, et al. Unique abnormalities in right ventricular longitudinal strain in systemic sclerosis patients. Circ Cardiovasc Imaging. 2016;9(6). 10.1161/CIRCIMAGING.115.003792. 10.1161/CIRCIMAGING.115.003792 PMC4902176 27266598

[12] Mukherjee M, Mercurio V, Tedford RJ, Shah AA, Hsu S, Mullin CJ, et al. Right ventricular longitudinal strain is diminished in systemic sclerosis compared with idiopathic pulmonary arterial hypertension. Eur Respir J. 2017;50(5):1701436. 10.1183/13993003.01436-2017 PMC5843490 29167303

[13] Mercurio V, Mukherjee M, Tedford RJ, Zamanian RT, Khair RM, Sato T, et al. Improvement in right ventricular strain with ambrisentan and tadalafil upfront therapy in scleroderma pulmonary arterial hypertension. Am J Respir Crit Care Med. 2018;197(3):388-91. 10.1164/rccm.201704-0789LE PMC5803650 28661697

[14] Kowal-Bielecka O, Avouac J, Pittrow D, Huscher D, Behrens F, Denton CP (2010). Echocardiography as an outcome measure in scleroderma-related pulmonary arterial hypertension: a systematic literature analysis by the EPOSS group. J Rheumatol.

[15] Preliminary criteria for the classification of systemic sclerosis (scleroderma). Subcommittee for scleroderma criteria of the American Rheumatism Association Diagnostic and Therapeutic Criteria Committee. Arthritis Rheum. 1980;23(5):581–90. 10.1002/art.1780230510 7378088

[16] van den Hoogen F, Khanna D, Fransen J, Johnson SR, Baron M, Tyndall A (2013). 2013 classification criteria for systemic sclerosis: an American College of Rheumatology/European League against Rheumatism collaborative initiative. Arthritis Rheum.

[17] Bissell LA, Anderson M, Burgess M, Chakravarty K, Coghlan G, Dumitru RB (2017). Consensus best practice pathway of the UK Systemic Sclerosis Study group: management of cardiac disease in systemic sclerosis. Rheumatology (Oxford).

[18] Olschewski H, Behr J, Bremer H, Claussen M, Douschan P, Halank M (2018). Pulmonary hypertension due to lung diseases: Updated recommendations from the Cologne Consensus Conference 2018. Int J Cardiol.

[19] Lang RM, Badano LP, Mor-Avi V, Afilalo J, Armstrong A, Ernande L (2015). Recommendations for cardiac chamber quantification by echocardiography in adults: an update from the American Society of Echocardiography and the European Association of Cardiovascular Imaging. Eur Heart J Cardiovasc Imaging.

[20] Rudski LG, Lai WW, Afilalo J, Hua L, Handschumacher MD, Chandrasekaran K (2010). Guidelines for the echocardiographic assessment of the right heart in adults: a report from the American Society of Echocardiography endorsed by the European Association of Echocardiography, a registered branch of the European Society of Cardiology, and the Canadian Society of Echocardiography. J Am Soc Echocardiogr.

[21] Berger M, Haimowitz A, Van Tosh A, Berdoff RL, Goldberg E (1985). Quantitative assessment of pulmonary hypertension in patients with tricuspid regurgitation using continuous wave Doppler ultrasound. J Am Coll Cardiol.

[22] Abbas AE, Fortuin FD, Schiller NB, Appleton CP, Moreno CA, Lester SJ (2003). A simple method for noninvasive estimation of pulmonary vascular resistance. J Am Coll Cardiol.

[23] Tello K, Wan J, Dalmer A, Vanderpool R, Ghofrani HA, Naeije R (2019). Validation of the tricuspid annular plane systolic excursion/systolic pulmonary artery pressure ratio for the assessment of right ventricular-arterial coupling in severe pulmonary hypertension. Circ Cardiovasc Imaging.

[24] Geyer H, Caracciolo G, Abe H, Wilansky S, Carerj S, Gentile F (2010). Assessment of myocardial mechanics using speckle tracking echocardiography: fundamentals and clinical applications. J Am Soc Echocardiogr.

[25] de Vet HC, Terwee CB, Knol DL, Bouter LM (2006). When to use agreement versus reliability measures. J Clin Epidemiol.

[26] Bland JM, Altman DG (1986). Statistical methods for assessing agreement between two methods of clinical measurement. Lancet..

[27] Norman GR, Stratford P, Regehr G (1997). Methodological problems in the retrospective computation of responsiveness to change: the lesson of Cronbach. J Clin Epidemiol.

[28] Portney LGWM (2009). Foundations of clinical research: applications to practice.

[29] Lahm T, Douglas IS, Archer SL, Bogaard HJ, Chesler NC, Haddad F (2018). Assessment of right ventricular function in the research setting: knowledge gaps and pathways forward. An Official American Thoracic Society Research Statement. Am J Respir Crit Care Med.

[30] Douglas PS, DeCara JM, Devereux RB, Duckworth S, Gardin JM, Jaber WA (2009). Echocardiographic imaging in clinical trials: American Society of Echocardiography Standards for echocardiography core laboratories: endorsed by the American College of Cardiology Foundation. J Am Soc Echocardiogr.

[31] Bunting KV, Steeds RP, Slater LT, Rogers JK, Gkoutos GV, Kotecha D (2019). A practical guide to assess the reproducibility of echocardiographic measurements. J Am Soc Echocardiogr.

[32] Shechtman O, Doi SAR, Williams GM (2013). The coefficient of variation as an index of measurement reliability. Methods of Clinical Epidemiology.

[33] Goransson C, Vejlstrup N, Scheike T, Carlsen J (2018). Implications of cardiac variability with cardiovascular magnetic resonance imaging for calculating trial sample size in pulmonary arterial hypertension. Int J Cardiol.

[34] Vonk Noordegraaf A, Westerhof BE, Westerhof N (2017). The relationship between the right ventricle and its load in pulmonary hypertension. J Am Coll Cardiol.

[35] Hinderliter AL, Willis PW, Barst RJ, Rich S, Rubin LJ, Badesch DB (1997). Effects of long-term infusion of prostacyclin (epoprostenol) on echocardiographic measures of right ventricular structure and function in primary pulmonary hypertension. Primary Pulmonary Hypertension Study Group. Circulation..

[36] Nath J, Demarco T, Hourigan L, Heidenreich PA, Foster E (2005). Correlation between right ventricular indices and clinical improvement in epoprostenol treated pulmonary hypertension patients. Echocardiography..

[37] Colalillo A, Grimaldi MC, Vaiarello V, Pellicano C, Leodori G, Gigante A, et al. In systemic sclerosis TAPSE/sPAP ratio can be used in addition to the DETECT algorithm for pulmonary arterial hypertension diagnosis. Rheumatology (Oxford). 2022;61(6):2450-2456. 10.1093/rheumatology/keab748 34605890

[38] Tello K, Axmann J, Ghofrani HA, Naeije R, Narcin N, Rieth A (2018). Relevance of the TAPSE/PASP ratio in pulmonary arterial hypertension. Int J Cardiol.

[39] Beaton DE, Bombardier C, Katz JN, Wright JG, Wells G, Boers M (2001). Looking for important change/differences in studies of responsiveness. OMERACT MCID Working Group. Outcome Measures in Rheumatology. Minimal Clinically Important Difference. J Rheumatol.

[40] Mathai SC, Puhan MA, Lam D, Wise RA (2012). The minimal important difference in the 6-minute walk test for patients with pulmonary arterial hypertension. Am J Respir Crit Care Med.

[41] Sato T, Tsujino I, Ohira H, Oyama-Manabe N, Ito YM, Takashina C (2017). Accuracy of echocardiographic indices for serial monitoring of right ventricular systolic function in patients with precapillary pulmonary hypertension. PLoS One.

[42] Mazurek JA, Vaidya A, Mathai SC, Roberts JD, Forfia PR (2017). Follow-up tricuspid annular plane systolic excursion predicts survival in pulmonary arterial hypertension. Pulm Circ.

[43] Argula RG, Karwa A, Lauer A, Gregg D, Silver RM, Feghali-Bostwick C (2017). Differences in right ventricular functional changes during treatment between systemic sclerosis-associated pulmonary arterial hypertension and idiopathic pulmonary arterial hypertension. Ann Am Thorac Soc.

[44] Hassoun PM, Zamanian RT, Damico R, Lechtzin N, Khair R, Kolb TM (2015). Ambrisentan and tadalafil up-front combination therapy in scleroderma-associated pulmonary arterial hypertension. Am J Respir Crit Care Med.

