# Supplementary material for: Defining minimal detectable difference in echocardiographic measures of right ventricular function in systemic sclerosis
Source: Arthritis Res Ther. 2022 Jun 18;24:146. doi: 10.1186/s13075-022-02835-5 (PMC9206258; doi:10.1186/s13075-022-02835-5)
Supplement: Supplementary file 1 — Additional file 1: Supplemental Table 1. Bootstrap estimated population minimal detectable differences for echocardiographic measurements. [file 13075_2022_2835_MOESM1_ESM.docx]

|  | Observed Coefficient | Bias | Bootstrap Std Error | 95% CI | |
| --- | --- | --- | --- | --- | --- |
| TAPSE | .10537165 | -.0023764 | .01904327 | .0680475  .0658143  .0745411 | .1426958(N)  .143795 (P)  .1553954(BC) |
| FAC | 2.9109725 | -.1125605 | .56019564 | 1.813009  1.695413  1.927821 | 4.008936(N)  3.863433(P)  4.048064(BC) |
| S’wave | 1.2701833 | -.0816464 | .48338367 | .3227687  .4969727  .5160428 | 2.217598(N)  2.115651(P)  2.193292(BC) |
| RVOT VTI | .81497671 | -.0185857 | .13729875 | .5458761  .54989  .608292 | 1.084077(N)  1.102572(P)  1.193429(BC) |
| Global RVLSS | 1.1424734 | -.0281674 | .19853962 | .7533429 .7521279  .8070588 | 1.531604(N)  1.533375(P)  1.607905(BC) |
| RVSP | 6.4619182 | -.6311198 | 2.5228052 | 1.517311  2.122028  2.267903 | 11.40653(N)  10.48203(P)  11.22358(BC) |

Supplemental Table 1.

Bootstrap Estimated Population Minimal Detectable Differences for Echocardiographic Measurements

Bootstrap estimates generated from entire cohort (N=40) with 1000 replications for select echocardiographic measurements. TAPSE: tricuspid annular plane systolic excursion; FAC: fractional area change; S’ wave: tissue Doppler of the tricuspid annulus S’ velocity; RVOT VTI: right ventricular outflow tract velocity time integral; Global RVLSS: RV longitudinal systolic speckle-derived strain; RVSP: right ventricular systolic pressure; N: normal confidence interval; P: percentile confidence interval; BC: bias-corrected confidence interval
